# Polygenic Risk Score Combined with Transcranial Sonography Refines Parkinson's Disease Risk Prediction

**DOI:** 10.1002/mdc3.70011

**Published:** 2025-02-28

**Authors:** Mart Kals, Anu Reigo, Maris Teder‐Laving, Mariliis Vaht, Estonian Biobank research team, Lili Milani, Reedik Mägi, Mari Nelis, Georgi Hudjašov, Tiit Nikopensius, Andres Metspalu, Toomas Toomsoo

**Affiliations:** ^1^ Estonian Genome Center, Institute of Genomics University of Tartu Tartu Estonia; ^2^ Institute of Molecular and Cell Biology University of Tartu Tartu Estonia; ^3^ Confido Medical Centre Tallinn Estonia; ^4^ School of Natural Sciences and Health Tallinn University Tallinn Estonia

**Keywords:** Parkinson's disease, polygenic risk score, substantia nigra hyperechogenicity, transcranial sonography

## Abstract

**Background:**

Dopaminergic neuron depletion in the substantia nigra (SN) and the pathological aggregation of α‐synuclein are the neuropathological hallmarks of Parkinson's disease (PD).

**Objectives:**

This study aimed to investigate the association between the polygenic risk score for PD (PD‐PRS) and transcranial sonography (TCS)‐measured SN hyperechogenicity to enhance the accuracy of PD susceptibility prediction.

**Methods:**

PD‐PRSs were calculated for over 41,000 Estonian Biobank participants age 55+ years without a PD diagnosis. Participants in the highest and lowest PD‐PRS percentiles (n = 222) underwent TCS measurements and Sniffin’ sticks olfactory testing. A multivariable logistic regression model was used to examine the associations between PD‐PRS, risk and prodromal markers, and SN hyperechogenicity.

**Results:**

Data from 204 participants with TCS measurements were analyzed, including 107 individuals in the high‐risk PD‐PRS group and 97 in the low‐risk PD‐PRS group. Incorporating PD‐PRS group assignment improved the explained variance in SN hyperechogenicity from 17.2% to 31.9%. Participants in the low‐risk PD‐PRS group had 0.16 times lower odds (95% confidence interval (CI) = 0.07–0.35, *P* < 0.001) of developing SN hyperechogenicity compared to high‐risk PD‐PRS individuals. Each unit increase in the Sniffin’ sticks olfactory test score was significantly associated with reduced odds of SN hyperechogenicity (adjusted odds ratio = 0.60, 95% CI = 0.47–0.78, *P* = 0.002).

**Conclusions:**

Our findings indicate that TCS‐measured SN hyperechogenicity is associated with PD‐PRS and olfactory impairment. This combined assessment may improve early diagnosis of prodromal PD by pinpointing individuals at increased risk.

Parkinson's disease (PD) is the second most common neurodegenerative disorder, affecting up to 3% of the population age 65+ years. The clinical diagnosis of PD relies on the presence of bradykinesia, muscular rigidity, and other motor features; however, patients with the disease also have multiple non‐motor symptoms that add to their overall disability.^1^ Epidemiological studies have identified potentially contributing environmental factors (eg, pesticide exposure) and prodromal comorbidities (eg, constipation, hyposmia, and depression) of PD.^2^, ^3^ Genetic testing has outlined that ~15% of PD cases are Mendelian, with pathogenic/likely pathogenic variants in *GBA1* (~10%), *LRRK2* (~3%), *PRKN* (~1%), and other PD genes (~1%).^4^, ^5^ Additionally, genome‐wide association studies (GWASs) have identified more than 90 risk loci as potential drivers of late‐onset PD development.^6^, ^7^ Some genetic markers are incorporated into the Movement Disorder Society (MDS) research criteria for prodromal PD, designated to identify individuals at an increased risk of developing PD.^8^, ^9^ Alternatively, polygenic risk scores (PRS) offer another approach to identify individuals with a high genetic predisposition to the disease.^10^

Transcranial sonography (TCS) has identified enlarged areas of echogenicity (hyperechogenicity) in the substantia nigra (SN) in patients with various neurodegenerative diseases, particularly PD. The Prospective Validation of Risk Factors for the Development of Parkinson Syndromes (PRIPS) study established SN hyperechogenicity (SN+) as an early marker that significantly increases the risk of PD.^11^, ^12^, ^13^ This finding holds potential to transform PD screening and early intervention strategies. Most studies describe the size of this TCS‐measured signal as a stable marker.^14^ Recent findings also suggest correlations between the TCS signal, disease severity, and dopamine transporter uptake.^15^

When combined with other risk factors, SN+ improves the sensitivity and specificity of PD detection.^16^, ^17^ A meta‐analysis showed that TCS‐based PD detection achieved 81% to 85% sensitivity and 85% to 88% specificity.^18^ However, the positive predictive value (PPV) of TCS in detecting PD is limited because of the mismatch between the prevalence of SN+ in the population and the proportion of individuals who develop PD. For instance, during the 3‐year follow‐up in the PRIPS study, SN+ as a single marker had a PPV of 3.1%.^19^ Notably, the PPV increased to 6.1% when combined with additional factors such as hyposmia and a family history of PD.

Alternatively, abnormalities detected by dopamine transporter imaging (DaTscan) serve as an important prodromal biomarker for PD. This technique enables the detection of early changes in the dopaminergic system^20^ and has been comprehensively validated in clinical trials.^21^ With sensitivity and specificity exceeding 90%,^22^ DaTscan demonstrates significant utility in identifying reduced putamen dopamine transporter binding even before clinical symptoms appear, highlighting its potential for early intervention and precise risk assessment.^23^ However, the routine clinical application of DaTscan remains limited because of its high cost and restricted availability.

Efforts have been made to incorporate non‐genetic factors into PD risk prediction algorithms.^24^, ^25^, ^26^, ^27^ In addition, several PRSs for PD (PD‐PRSs) have been developed to study associations with established disease.^28^, ^29^, ^30^, ^31^, ^32^, ^33^ A PD‐PRS alone is not sufficiently specific to have diagnostic value, but the addition of TCS‐detected echogenicity results for individuals with the highest polygenic risk could enable combined risk effect estimation with diagnostic value long before clinical symptoms appear. In this proof‐of‐concept study, we evaluated the association between TCS‐measured SN echogenicity and PD‐PRS among participants of the Estonian Biobank (EstBB).^34^ This combined approach has potential to improve the prediction of PD susceptibility. The analysis specifically targeted individuals with high and low PD‐PRS who had not yet been diagnosed with PD.

## Methods

### Study Cohort

The population‐based EstBB contains data for 213,000 inhabitants of Estonia (~20% of the adult population). For the participants, a comprehensive questionnaire (providing objective information, and information on, eg, physical activity and diet) is filled out and DNA, plasma, and white blood cell samples are stored. The database is updated by regular linkage to the national health databases. The EstBB project is being conducted according to the Estonian Human Genes Research Act, and all participants have signed a broad informed consent form.^34^

### Study Design and Participants

The inclusion criteria for the EstBB participants were: (1) no evidence of PD based on medical history records in the national electronic health database; (2) current age ≥55 years and alive; and (3) Estonian nationality and residence in Estonia. PRSs were calculated for all individuals who met the inclusion criteria (n = 41,042, mean age = 66.4 [standard deviation (SD) = 8.7] years, 69.3% females). Approximately equal numbers of female and male participants from the highest and lowest PD‐PRS percentiles were selected using stringent cutoff values (<0.5th and >99.5th percentiles for females, <1st and >99th percentiles for males).

In total, 244 individuals (136 females) were assigned to the high PD‐PRS group, and 268 individuals (137 females) were assigned to the low PD‐PRS group. Invitations were sent out in batches to target ~100 participants per PD‐PRS group. Overall, 447 invitations were distributed, inviting individuals to attend a single‐day appointment with a neurologist at one of three centers in Estonia (Tallinn, Tartu, and Kuressaare) between November 2021 and June 2022.

### Polygenic Risk Score Calculation

PRSs were calculated using PRS‐CS‐auto (version 2021‐01‐04),^35^ which infers posterior variant effect sizes under continuous shrinkage priors. This method is based on GWAS summary statistics and uses an external linkage disequilibrium reference panel, specifically 503 European samples from the 1000 Genomes Project. GWAS summary statistics from the largest‐known meta‐analysis of PD^6^ (40,150 cases and 751,098 controls independent of EstBB participants) were used to infer genome‐wide genotypes for PD‐PRS calculation. The PD‐PRS variable was dichotomized using the upper and lower percentiles for subsequent analyses (Fig. S1).

### Transcranial Sonography

To ensure the reproducibility and reliability of measurements, TCS examinations for each participant were conducted by an experienced sonographer, who was blinded to PD‐PRS group allocation, neurological assessments, and other comorbidities for all participants. Standard brainstem sonography was conducted through the preauricular acoustic bone window using a 2.5‐MHz S4‐2 FAST transducer in two‐dimensional mode with the ZST+ platform (Mindray; Shenzhen, China). After identifying the butterfly‐shaped mesencephalic brainstem, the maximal ipsilateral SN area was located in the axial plane. The image was then captured, manually labeled, and planimetry was performed (Fig. S2).

SN echogenicity was assessed independently from the right and left temporal windows. The echogenic area was calculated as the arithmetic mean of three repeated measurements for each side, and the higher mean value was used as the final measure of echogenicity, considering the situation of SN+ detection on only one side (Fig. S3). Individuals with deficient bone mineral density were excluded from the study. The SN+ area was defined using a cutoff value of ≥0.22 cm^2^ as the marked SN+, determined based on previous empirical evidence from 49 patients with PD and 50 healthy controls using the Mindray ultrasound system. The control group (mean age = 60.7 [SD = 5.9] years, 44.9% females) was matched to the PD group (mean age = 60.5 [SD = 4.6] years, 40.8% females) to reflect the typical age of PD onset. At a threshold value of 0.22 cm^2^, sensitivity was 100% and specificity was 96%, with a PPV of 96% and a negative predictive value of 100%.

### Clinical Assessment

A neurologist blinded to PD‐PRS group allocation interviewed the study participants and conducted neurological assessments. During the interview, the participants were questioned about the most common PD risk factors and comorbidities, including heart, lung, thyroid diseases, diabetes, and migraine (with or without aura). None of the participants had undergone polysomnography to formally diagnose rapid eye movement (REM) sleep behavior disorder. However, participants were asked about disturbing dreams and abnormal limb movements that disturbed their bed partner's sleep. These symptoms are referred to as dream enactment behavior (DEB) in this study.

To assess depression, participants were asked whether they had ever taken or were currently taking antidepressants and whether these medications had been prescribed specifically for symptoms of depression. A comprehensive mental state examination was not conducted.

Olfactory sensitivity was assessed using the 12‐item Sniffin’ Sticks test (Heinrich Burghart GmbH, Wedel, Germany),^36^ which has been successfully adapted for cross‐cultural use.^37^ The total score ranges from 0 to 12, with hyposmia typically defined by scores below 10 and anosmia by scores below 6.

PD was diagnosed based on established clinical criteria^38^ with a particular focus on the presence of motor symptoms indicative of the disease.

### Genotyping and Imputation

EstBB samples were genotyped at the Core Genotyping Lab of the Institute of Genomics, University of Tartu, using Illumina (San Diego, CA) global screening arrays v1.0, v2.0, and v2.0_EST. Individuals whose sex defined by chromosome X heterozygosity did not match the sex recorded in phenotype data were excluded from the analysis. Before genotype imputation, variants were filtered by call rate <95%, Hardy–Weinberg equilibrium *P* < 1 × 10^−4^ (autosomal variants only), and minor allele frequency <1%. Prephasing was performed using Eagle^39^ software (version 2.3), and imputation was performed using Beagle^40^ (version 28Sep18.793), with the use of an Estonian population–specific imputation reference panel built from 2297 whole‐genome sequencing samples.^41^

### Statistical Analysis

The demographic and clinical characteristics of the study cohort were analyzed after stratification by extreme PD‐PRS (high vs. low) and SN echogenicity (≥0.22 cm^2^ vs. <0.22 cm^2^) groups. Quantitative variables were compared between groups using the *t* test, whereas categorical variables were analyzed with Fisher's exact test. Associations between Sniffin’ sticks score, age, and SN echogenicity groups were assessed using Pearson's $\chi^{2}$ test.

To evaluate the relationship between PD‐PRS, various risk factors, and prodromal markers in relation to SN+ status, a multivariable logistic regression model was applied. Only categorical variables with expected frequencies of 10 or more in all cells of the contingency table were included in the model. Backward elimination was used to refine the model. Adjusted odds ratios (aORs) for SN+ development with 95% confidence intervals (CIs) are reported.

The performance of the multivariable model was assessed using Nagelkerke's pseudo‐*R* ^2^ and the area under the receiver operating characteristics curve (AUC). Sensitivity analyses were performed to explore the effect of baseline and prodromal markers, as well as PD‐PRS, on SN+ status, excluding individuals with newly diagnosed PD.

Two‐sided *P*‐values <0.05 were considered statistically significant. All statistical analyses were performed using R software (version 4.2.2; R Core Team, 2013).

## Results

Of the 447 individuals who met the inclusion criteria and were invited to participate, 222 agreed to take part in the study, yielding a response rate of 49.7%. However, 18 (8%) participants were excluded from the analysis because of insufficient TCS‐measured acoustic bone windows. The final sample included 204 participants: 107 in the high‐risk PD‐PRS group (mean age = 64.3 [SD = 7.3] years, 47.7% females) and 97 in the low‐risk PD‐PRS group (mean age = 65.3 [SD = 7.0] years, 46.4% females). The participants' demographic characteristics and clinical data collected during the neurologist visits stratified by PD‐PRS groups are summarized in Table 1.

**TABLE 1:** Demographic and clinical characteristics of individuals in high and low polygenetic risk groups for PD

	High^a^ PD‐PRS (n = 107)	Low^a^ PD‐PRS (n = 97)	*P*‐value
Age (SD, y)	64.3 (7.3)	65.3 (7.0)	0.325
Sex (%)			0.967
Female	51 (47.7)	45 (46.4)	
Male	56 (52.3)	52 (53.6)	
Family history of PD (%)	10 (9.3)	8 (8.2)	0.977
Area of echogenicity in SN (SD, cm^2^)	0.21 (0.09)	0.15 (0.06)	<0.001
Occurrence of SN+^b^ (%)	46 (43.0)	10 (10.3)	<0.001
Newly diagnosed PD (%)	9 (8.4)	3 (3.1)	0.189
Smoking (%)			0.062
Never smoker	64 (59.8)	71 (73.2)	
Former/current smoker	43 (40.2)	26 (26.8)	
Sniffin’ sticks total score (SD)	9.46 (1.85)	9.73 (1.19)	0.224
Hyposmia (%)	43 (41.3)	37 (38.1%)	0.750
Anosmia (%)	5 (4.8%)	0 (0)	0.083
Coffee (SD, cups a day)	2.16 (1.49)	1.85 (1.14)	0.095
Tea (SD, cups a day)	1.02 (1.20)	0.77 (0.88)	0.100
Dream enactment behavior (%)	5 (4.7)	13 (13.4)	0.051
Constipation (%)	16 (15.0)	11 (11.3)	0.580
Depression (%)	20 (19.0)	27 (27.8)	0.190
Diabetes (%)	8 (7.5)	11 (11.3)	0.480
Migraine with aura (%)	10 (9.3)	2 (2.1)	0.056
Migraine without aura (%)	7 (6.5)	9 (9.3)	0.642
Use of blood pressure medication (%)	45 (42.1)	54 (55.7)	0.071
Use of statin (%)	26 (24.3)	35 (36.1)	0.092
Use of anticoagulants (%)	7 (6.5)	15 (15.5)	0.068
Use of diabetes medication (%)	7 (6.5)	7 (7.2)	1.000

^a^ Participants were assigned to the high and low PD‐PRS groups based on the upper and lower percentiles of the PD‐PRS, with cutoff values set at 0.5% for females and 1% for males.

^b^ SN echogenicity area ≥0.22 cm^2^.

Abbreviations: PD, Parkinson's disease; PRS, polygenic risk score; SD, standard deviation; SN, substantia nigra.

**FIG. 1:**
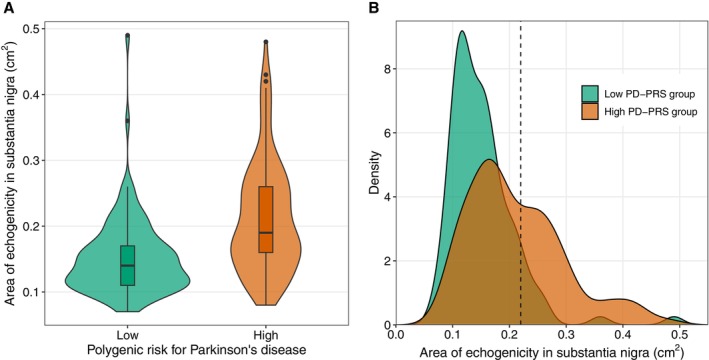
Distribution of substantia nigra echogenicity area (cm^2^) among individuals with high (n = 107) and low (n = 97) polygenic risk scores for Parkinson's disease (PD‐PRS). (**A**) A violin plot illustrating the distribution within high and low PD‐PRS groups, with median values of 0.19 (interquartile range [IQR] = 0.10) and 0.14 (IQR = 0.06), respectively. (**B**) A density plot showing the mean echogenicity values for the high and low PD‐PRS groups: 0.21 (standard deviation [SD] = 0.09) and 0.15 (SD = 0.06), respectively. The dashed line indicates the threshold for substantia nigra hyperechogenicity, set at 0.22 cm^2^.

In the high‐risk PD‐PRS group, 10 (9.3%) participants had a family history of PD, compared to eight (8.2%) participants in the low‐risk PD‐PRS group (*P* = 0.977). Depression was the most prevalent comorbidity, affecting 19.0% of individuals in the high‐risk group and 27.8% in the low‐risk group (*P* = 0.190). The most divergent comorbidities between the high‐ and low‐risk groups included migraine with aura (9.3% vs. 2.1%, *P* = 0.056) and DEB (4.7% vs. 13.4%, *P* = 0.051). Blood pressure, cholesterol levels, and the use of diabetes medication were similar between the two groups. Nine (8.4%) participants in the high‐risk group were diagnosed with PD, including two who had previously been suspected of having PD and were referred to neurologists. In the low PD‐PRS group, three (3.1%) individuals were newly diagnosed with PD. The most significant difference between the high‐ and low‐risk groups was in SN echogenicity, with mean values of 0.21 and 0.15 cm^2^, respectively (*P* < 0.001) (Fig. 1). SN values ≥0.22 cm^2^ were observed in 43.0% of individuals in the high‐risk group compared to 10.3% of the low‐risk group (*P* < 0.001).

When the cohort was stratified by SN echogenicity (≥0.22 cm^2^ vs. <0.22 cm^2^), the pattern of comorbidities and family history of PD were similar between the two groups, except that migraine with aura was significantly more prevalent in the SN+ group (16.1% vs. 2.0%, *P* = 0.001). The SN+ group also included a higher proportion of individuals with a high PD‐PRS risk (82.1% vs. 41.2%, *P* < 0.001) and demonstrated poorer olfactory test results (mean total Sniffin’ sticks score: 8.70 vs. 9.92, *P* < 0.001). The Sniffin’ sticks score also declined with increasing age (*P* < 0.001) (Fig. S4). Notably, all 12 newly diagnosed PD patients were observed in the SN+ group (Table 2).

**TABLE 2:** Demographic and clinical characteristics of individuals with and without SN hyperechogenicity (SN echogenicity area ≥0.22 cm^2^ vs. SN echogenicity area <0.22 cm^2^)

	SN^a^ ≥ 0.22 cm^2^ (n = 56)	SN^a^ < 0.22 cm^2^ (n = 148)	*P*‐value
Age (SD, y)	65.5 (6.8)	64.6 (7.3)	0.429
Sex (%)			0.127
Female	21 (37.5)	75 (50.7)	
Male	35 (62.5)	73 (49.3)	
Family history of PD (%)	5 (8.9)	13 (8.8)	1
PD‐PRS risk (%)			<0.001
High	46 (82.1)	61 (41.2)	
Low	10 (17.9)	87 (58.8)	
Newly diagnosed PD (%)	12 (21.4)	0 (0.0)	<0.001
Sniffin’ sticks total score (SD)	8.70 (1.83)	9.92 (1.33)	<0.001
Hyposmia (%)	33 (61.1)	47 (32.0)	<0.001
Anosmia (%)	4 (7.4)	1 (0.7)	0.028
Dream enactment behavior (%)	4 (7.1)	14 (9.5)	0.807
Constipation (%)	7 (12.5)	20 (13.5)	1
Depression (%)	9 (16.4)	38 (25.9)	0.217
Diabetes (%)	4 (7.1)	15 (10.1)	0.699
Migraine with aura (%)	9 (16.1)	3 (2.0)	0.001

^a^ SN echogenicity area.

Abbreviations: SN, substantia nigra; SD, standard deviation; PD, Parkinson's disease; PRS, polygenic risk score.

**FIG. 2:**
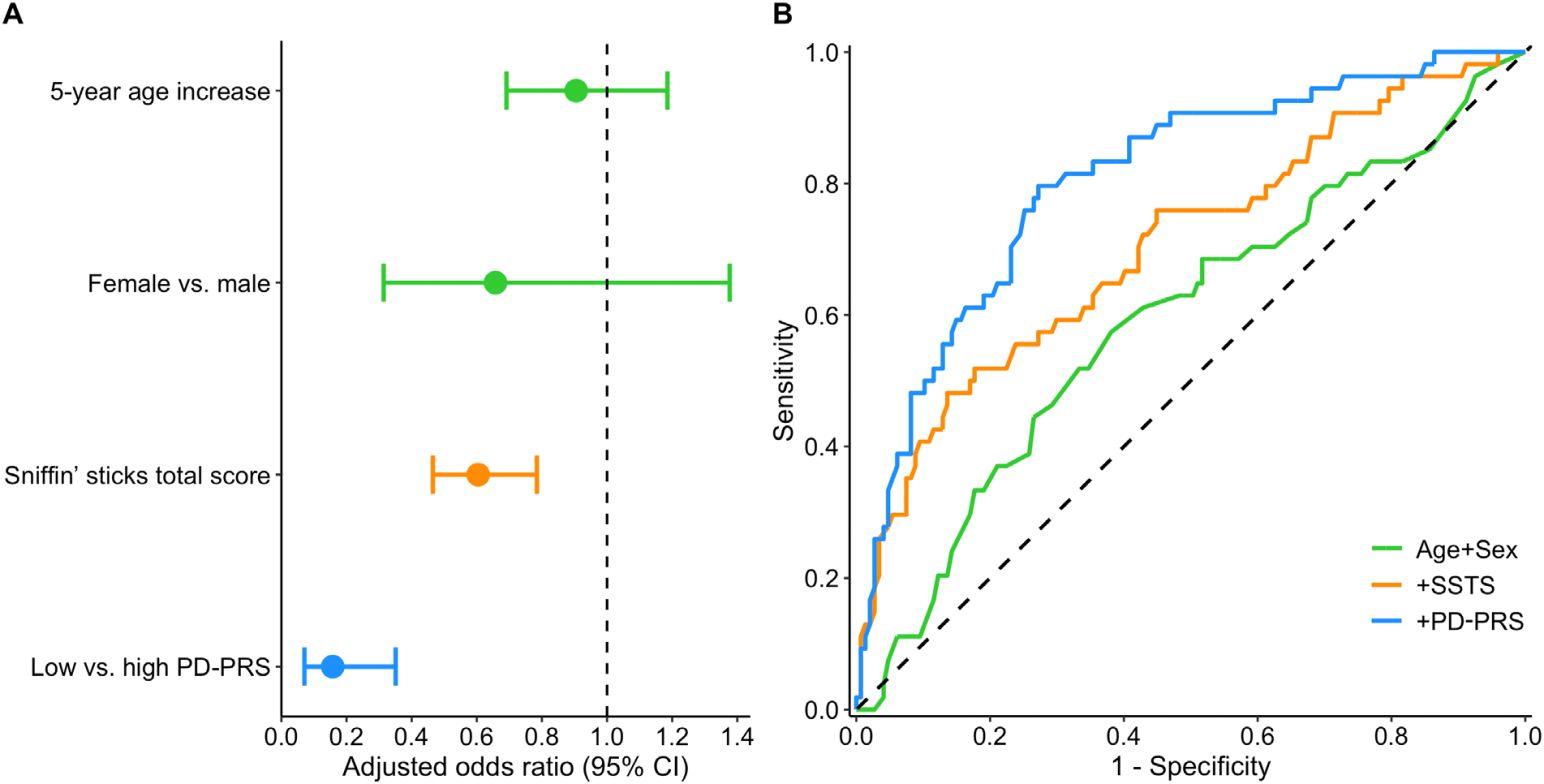
Predictive models for substantia nigra hyperechogenicity (area ≥0.22 cm^2^). (**A**) Adjusted odds ratios with 95% confidence intervals (CIs). (**B**) Receiver operating characteristic curves for three models: (1) age + sex (area under the curve [AUC] = 0.592); (2) age + sex + Sniffin’ sticks total score (SSTS; AUC = 0.706); and (3) age + sex + SSTS + polygenic risk score for Parkinson's disease (PD‐PRS; AUC = 0.807).

The multivariable logistic regression analysis showed that each 1‐point increase in the Sniffin’ sticks score was significantly associated with a decrease in the odds of SN+ (aOR = 0.58, 95% CI = 0.46–0.75, *P* < 0.001). The model explained 17.2% of the variance in the SN measure (AUC = 0.706) (Table 3, model 1). When the dichotomized PD‐PRS variable was added, the model performed significantly better (likelihood ratio test, *P* < 0.001), explaining 31.9% of the variance in the SN measure (AUC = 0.807) (Table 3, model 2). The odds of SN+ were significantly lower in the low‐risk PD‐PRS group compared to the high‐risk group (aOR = 0.16, 95% CI = 0.07–0.35, *P* < 0.001), and the olfactory test result remained significant (aOR = 0.60, 95% CI = 0.47–0.78, *P* = 0.002). The findings of the multivariable analysis are presented in Figure 2. No significant interactions were detected between the PD‐PRS group and age (*P* = 0.337), sex (*P* = 0.816), or total Sniffin’ sticks score (*P* = 0.576). Sensitivity analyses, excluding the 12 newly diagnosed PD patients, showed consistent results (Tables S1 and S2).

**TABLE 3:** Associations between substantia nigra hyperechogenicity and independent predictors as determined by multivariable logistic regression

	OR (95% CI)	*P*‐value
Model 1		
Age (5‐y increase)	0.86 (0.67–1.11)	0.248
Sex: female	0.74 (0.37–1.48)	0.394
Sniffin’ sticks total score	0.58 (0.46–0.75)	<0.001
*R* ^*2*^ = 17.2%, AUC = 0.706 (95% CI = 0.621–0.791)		
Model 2		
Age (5‐y increase)	0.91 (0.69–1.19)	0.469
Sex: female	0.66 (0.31–1.38)	0.266
Sniffin’ sticks total score	0.60 (0.47–0.78)	0.002
PD‐PRS risk: low	0.16 (0.07–0.35)	<0.001
*R* ^*2*^ = 31.9%, AUC = 0.807 (95% CI = 0.739–0.875); likelihood‐ratio test of model 2 vs. model 1, *P* < 0.001.		

Abbreviations: OR, odds ratio; CI, confidence interval; AUC, area under the receiver operating characteristic curve; PD, Parkinson's disease; PRS, polygenic risk score.

The 12 newly diagnosed PD patients had significantly larger mean areas of SN echogenicity compared to all other participants (0.29 vs. 0.18 cm^2^, *P* < 0.001) and lower olfactory test scores (8.08 vs. 9.69, *P* < 0.001) (Table 4). Although the sample size was small and the differences were not statistically significant, the newly diagnosed PD group included a higher proportion of men (75.0% vs. 51.6%, *P* = 0.143) and individuals with high‐risk PD‐PRS (75.0% vs. 51.0%, *P* = 0.189) compared with those without a PD diagnosis.

**TABLE 4:** Demographic and clinical characteristics of individuals with newly diagnosed PD

	Newly diagnosed PD (n = 12)	No PD (n = 192)	*P*‐value
Age (SD, y)	66.75 (6.02)	64.68 (7.23)	0.333
Sex (%)			0.143
Male	9 (75.0)	99 (51.6)	
Female	3 (25.0)	93 (48.4)	
PD‐PRS risk (%)			0.189
High	9 (75.0)	98 (51.0)	
Low	3 (25.0)	94 (49.0)	
Sniffin’ sticks total score (SD)	8.08 (1.38)	9.69 (1.54)	0.001
Hyposmia (%)	10 (83.3)	70 (37.0)	0.004
Anosmia (%)	0 (0)	5 (2.6)	1
Area of echogenicity in SN (SD, cm^2^)	0.29 (0.07)	0.18 (0.08)	<0.001
Occurrence of SN+^a^ (%)	12 (100)	44 (22.9)	<0.001
Dream enactment behavior (%)	1 (8.3)	17 (8.9)	1
Constipation (%)	3 (25.0)	24 (12.5)	0.201
Depression (%)	2 (16.7)	45 (23.7)	0.736
Diabetes (%)	2 (16.7)	17 (8.9)	0.310
Migraine with aura (%)	4 (33.3)	8 (4.2)	0.003

^a^ SN echogenicity area ≥0.22 cm^2^.

Abbreviations: PD, Parkinson's disease; SD, standard deviation; PRS, polygenic risk score; SN, substantia nigra.

## Discussion

Previous studies have outlined various demographic and clinical risk factors (eg, sex, age, family history of PD, and SN+) along with prodromal markers (REM sleep behavior disorder, constipation, olfactory impairment, and depression) associated with a differential risk of PD development.^42^ Although PD‐PRS is a meaningful tool for investigating the polygenic component of PD,^43^ our study established a significant association between PD‐PRS and TCS‐measured SN+. We demonstrated that individuals with high PD‐PRS risk had significantly larger areas of SN echogenicity compared to those with low risk (0.21 vs. 0.15 cm^2^, *P* < 0.001). This combined approach shows promise for clinical application, especially in identifying candidates for early intervention. However, aside from differences in SN echogenicity, the groups stratified by PD‐PRS were similar in characteristics such as PD incidence, family history of PD, and prodromal markers.

SN+ has been identified as one among several key markers for predicting the onset of PD.^15^ Although it plays an important role in assessing PD risk, its predictive accuracy is strengthened when combined with other factors, such as genetic profiles or clinical assessments. A threshold value of 0.22 cm^2^ was used to define marked SN+. In univariate analyses, SN+ was found to be associated with PD‐PRS–based risk, olfactory loss, migraine with aura, and PD incidence. Previous studies have shown that TCS can detect SN+ (both moderate and marked) in ~83% of PD patients, even in the very early stages of the disease.^18^ However, ~13% of healthy individuals also show SN+, indicating a reduced specificity of SN+ as a standalone diagnostic marker.^18^ In this study, all 12 newly diagnosed PD patients had SN echogenicity areas exceeding the 0.22 cm^2^ threshold. However, approximately a quarter of participants without a PD diagnosis also exhibited SN+, highlighting the need for cautious interpretation. Proper skills and experience are needed for accurate SN+ assessment, and it should always be combined with other risk indicators, as recommended by the MDS research and clinical diagnostic criteria for prodromal PD.^8^, ^38^

In line with the hypothesis that PD susceptibility, as determined by PD‐PRS, is mediated by SN+, our multivariate analysis showed that individuals with the highest PD‐PRS risk had nearly six times greater odds of exhibiting SN+ compared to those with the lowest PD‐PRS risk. This finding, beyond the established risk factors,^44^ provides additional evidence for the genetic predisposition to SN+ as suggested by previous studies.^45^, ^46^, ^47^ Therefore, the presence of SN+ has been observed in carriers of disease‐causing variants even before functional neuroimaging detects dopaminergic system abnormalities,^45^ suggesting that SN+ arises before neurodegeneration. Consequently, the SN+ can indeed be considered as an early risk marker rather than a sign of prodromal status. The inclusion of PD‐PRS in our analysis increased the explained variance in SN+ from 17.2% to 31.9%. These findings suggest that the risk of developing PD is, at least in part, mediated by genetic factors associated with SN+.

Population‐based cohort studies have shown that individuals with hyposmia, a common prodromal manifestation of PD, are at increased risk of developing PD within a few years.^48^, ^49^ We demonstrated that individuals with better olfactory sensitivity had a reduced risk of developing SN+, supporting recent finding that links PD‐PRS with olfactory impairment.^50^

Additionally, SN+, hyposmia, and mild parkinsonian signs (MPS) have been linked to an elevated risk of developing PD over a 5 to 10‐year follow‐up period, with SN+ and hyposmia identified as the most robust and consistent predictors.^13^, ^51^ Notably, both SN+ and hyposmia also predicted the development of MPS in the general elderly population, not just in individuals with PD,^52^ suggesting that these markers may reflect general nigral vulnerability or dysfunction, rather than being specific to PD pathology. However, their PPV remains relatively low when used alone, underscoring the need for multi‐marker approaches to improve the specificity of PD prediction. Additionally, incorporating α‐synuclein seed amplification assays could further enhance diagnostic accuracy in identifying the prodromal phase of PD, aligning with recent advancements in the biological classification of PD^53^, ^54^ and supporting their inclusion in future predictive models.

Array‐based genotyping, GWAS analysis, and subsequent PRS calculations are well suited for large‐scale population studies aimed at early identification of disease risk and potential treatment responses. By identifying individuals with the highest PRS, targeted follow‐up and additional tests could improve diagnostic accuracy, aiding in the differentiation between affected individuals and healthy ones. This approach could also reduce the costs associated with primary intervention.

The main strength of this study is its use of a large, population‐based EstBB cohort, which is regularly updated by linkage to electronic health records and represents ~20% of the adult population in Estonian. This resource allowed us to identify and re‐contact individuals age 55+ years without a PD diagnosis, achieving a response rate of ~50%. Moreover, imputed genome‐wide array data for PRS calculation were available for all EstBB participants, allowing us to incorporate PD‐PRS alongside non‐genetic predictors in our multivariate statistical analysis.

However, this study has several limitations. First, the PD‐PRS calculation is currently applicable only to individuals genetically similar to the European reference population, and large‐scale trans‐ethnic GWAS studies and target cohorts are needed to expand the transferability of these results to other populations. Additionally, during the neurologist appointment, study participants did not undergo essential clinical procedures, such as polysomnography for REM sleep assessment or comprehensive mental health evaluations. Consequently, we relied on self‐reported prodromal comorbidities, which may differ from findings in studies that used confirmed diagnoses. Ideally, DaTscan and TCS could complement each other in the diagnostic process. TCS could fulfill the role of primary screening because of its cost‐effectiveness and availability, whereas DaTscan could be recommended to confirm diagnoses in more complex cases. Future research should focus on standardizing TCS protocol and improving its diagnostic accuracy to maximize its clinical utility.^55^ Last, the cross‐sectional design of our study lacks the ability to assess the prognostic value for PD incidence or explore its relationships with clinical and prodromal biomarkers. This issue will be addressed in a follow‐up study.

In conclusion, this study provides new evidence demonstrating an association between PD‐PRS and SN+ in apparently healthy individuals, emphasizing the value of these markers in understanding PD susceptibility. Incorporating additional risk factors and prodromal markers, such as olfactory impairment, increases the predictive power of models being tested. This study suggests the potential to improve detection of individuals at risk for PD by integrating PD‐PRS, olfactory testing, dopaminergic deficits (DaTscan), and clinical assessments. Future research should focus on optimally combining these markers to facilitate early PD identification and address its complex nature and clinical phenotypic heterogeneity for personalized care.

## Author Roles

(1) Research project: A. Conception, B. Organization, C. Execution; (2) Statistical Analysis: A. Design, B. Execution, C. Review and Critique; (3) Manuscript Preparation: A. Writing of the First Draft, B. Review and Critique

M.K.: 1A, 1B, 2A, 2B, 3A, 3B

A.R.: 1B, 3B

M.T.L.: 1B, 3B

M.V.: 1A, 1B, 3B

T.N.: 2C, 3A, 3B

A.M.: 1A, 1B, 2A, 2C, 3B

T.T.: 1A, 1B, 1C, 2A, 2C, 3A, 3B

## Disclosures

**Ethical Compliance Statement:** This study was approved by the Estonian Council on Bioethics and Human Research (no. 1.1–12/185). Written informed consent was obtained from all participants. We confirm that we have read the Journal's position on issues involved in ethical publication and affirm that this work is consistent with those guidelines.

**Funding Sources and Conflict of Interest:** This research was supported by Tallinn University (research grant no. TF3720 to T.T.), the Ministry of Education and Research Centres of Excellence grant TK214 (Centre of Excellence for Personalized Medicine), and the European Union through the European Regional Development Fund Project (no. 2014–2020.4.01.15–0012 GENTRANSMED). The authors declare no conflicts of interest relevant to this work.

**Financial Disclosures for the Previous 12 Months:** The authors declare that there are no additional disclosures to report.

## Supporting information


**Figure S1.** Distribution of standardized polygenic risk score for Parkinson's disease (PD‐PRS) among Estonian Biobank participants meeting the study inclusion criteria (n = 41,042). Among them, 244 individuals were assigned to the high‐risk PD‐PRS group (top percentiles, shown in red), and 268 to the low‐risk PD‐PRS group (bottom percentiles, shown in green).

**Figure S2.** Transcranial sonographic images of mesencephalic brain stems. (A) Image showing an individual with substantia nigra (SN) hyperechogenicity. (B) Image showing an individual without SN hyperechogenicity. The boundaries of the SN echogenicity areas were manually outlined, and numerical measurements were obtained using the TCS machine (ZST+ platform).

**Figure S3.** Echogenicity areas of the substantia nigra (SN) measured from left and right temporal windows, stratified by polygenic risk score for Parkinson's disease (PD‐PRS) groups. The mean echogenicity values are 0.16 cm^2^ (standard deviation [SD]: 0.07) for the left side, 0.17 cm^2^ (SD: 0.08) for the right side, and 0.18 cm^2^ (SD: 0.08) for the maximum of both sides. A correlation analysis shows a strong association between left and right side measurements (Pearson's *r* = 0.80, *P* < 0.001). The solid line represents a slope of 1, with dotted lines marking ±0.05 cm^2^ intervals. The red dashed lines indicate the SN hyperechogenicity threshold value of 0.22 cm^2^.

**Figure S4.** Distribution of Sniffin’ sticks total score by age and substantia nigra (SN) echogenicity. Sniffin’ sticks scores decrease with advancing age and are significantly lower in individuals with SN hyperechogenicity (Pearson's $\chi^{2}$ test, both *P* < 0.001). Error bars represent ± standard deviations, illustrating the uncertainty in measurements.

**Table S1.** Sensitivity analysis excluding individuals with newly diagnosed Parkinson's disease (PD). Twelve individuals newly diagnosed with PD were excluded from the analysis. Participants were compared based on the presence (substantia nigra (SN) echogenicity area ≥0.22 cm^2^) or absence (SN echogenicity area <0.22 cm^2^) of SN hyperechogenicity.

**Table S2.** Multivariable logistic regression analysis for substantia nigra (SN) hyperechogenicity. The analysis excludes 12 individuals with newly diagnosed Parkinson's disease, comparing participants with SN hyperechogenicity (SN echogenicity area ≥0.22 cm^2^, n = 44) and those without (SN echogenicity area <0.22 cm^2^, n = 148).

## Data Availability

The individual‐level data from the Estonian Biobank are available under restricted access and can only be obtained with approval from the Estonian Council on Bioethics and Human Research.

## Appendix

Estonian Biobank research team: Lili Milani, Reedik Mägi, Mari Nelis, Georgi Hudjašov.
