# Supplementary figures and images for: Polygenic Risk Score Combined with Transcranial Sonography Refines Parkinson's Disease Risk Prediction
Source: Mov Disord Clin Pract. 2025 Feb 28;12(7):928–37. doi: 10.1002/mdc3.70011 (PMC12274997; doi:10.1002/mdc3.70011)

Females ( $n = 28,445$ )

Percentiles: ■ <0.5% ■ 0.5–99.5% ■ >99.5%

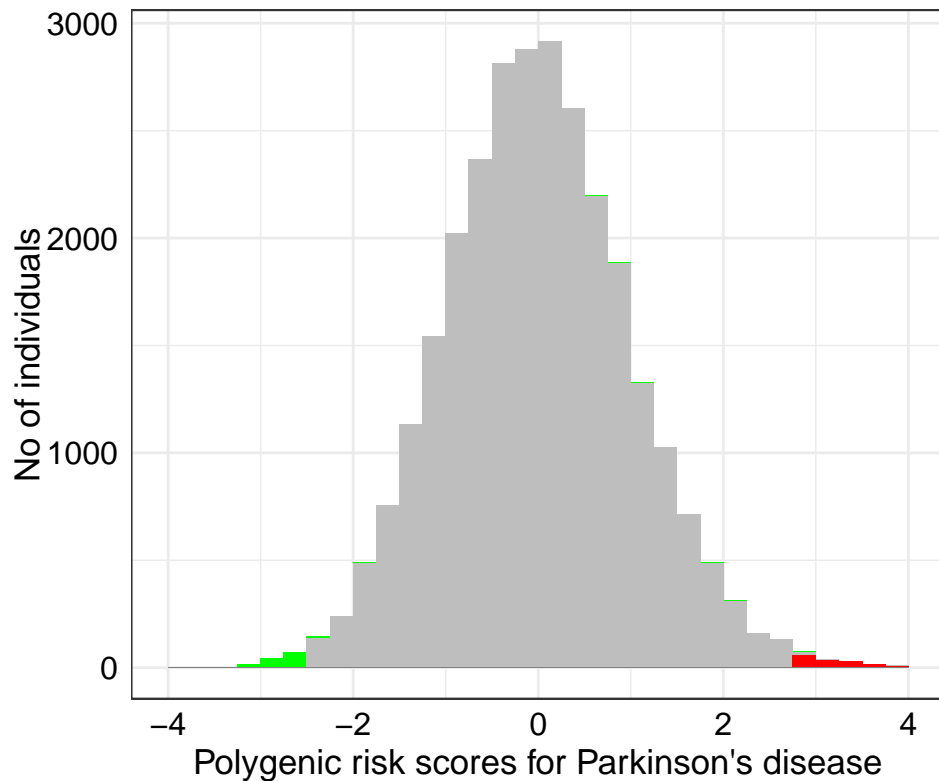

Males ( $n = 12,597$ )

Percentiles: ■ <1% ■ 1–99% ■ >99%

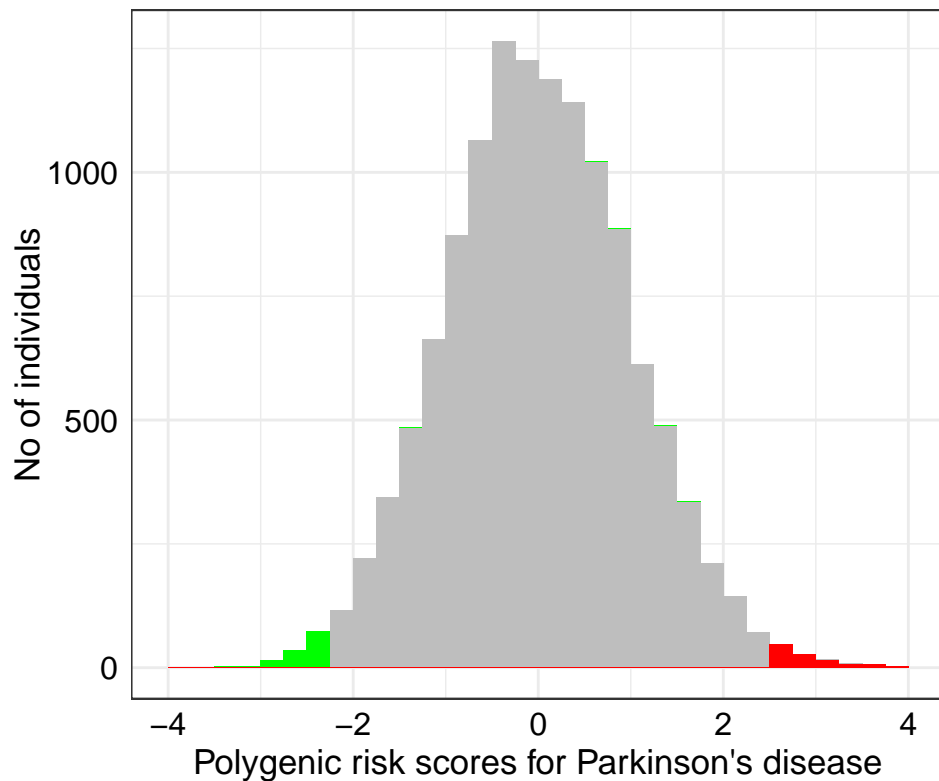

Supplement: Supplementary file 1 — Figure S1. Distribution of standardized polygenic risk score for Parkinson's disease (PD‐PRS) among Estonian Biobank participants meeting the study inclusion criteria (n = 41,042). Among them, 244 individuals were assigned to the high‐risk PD‐PRS group (top percentiles, shown in red), and 268 to the low‐risk PD‐PRS group (bottom percentiles, shown in green). [file MDC3-12-928-s002.pdf]

**A**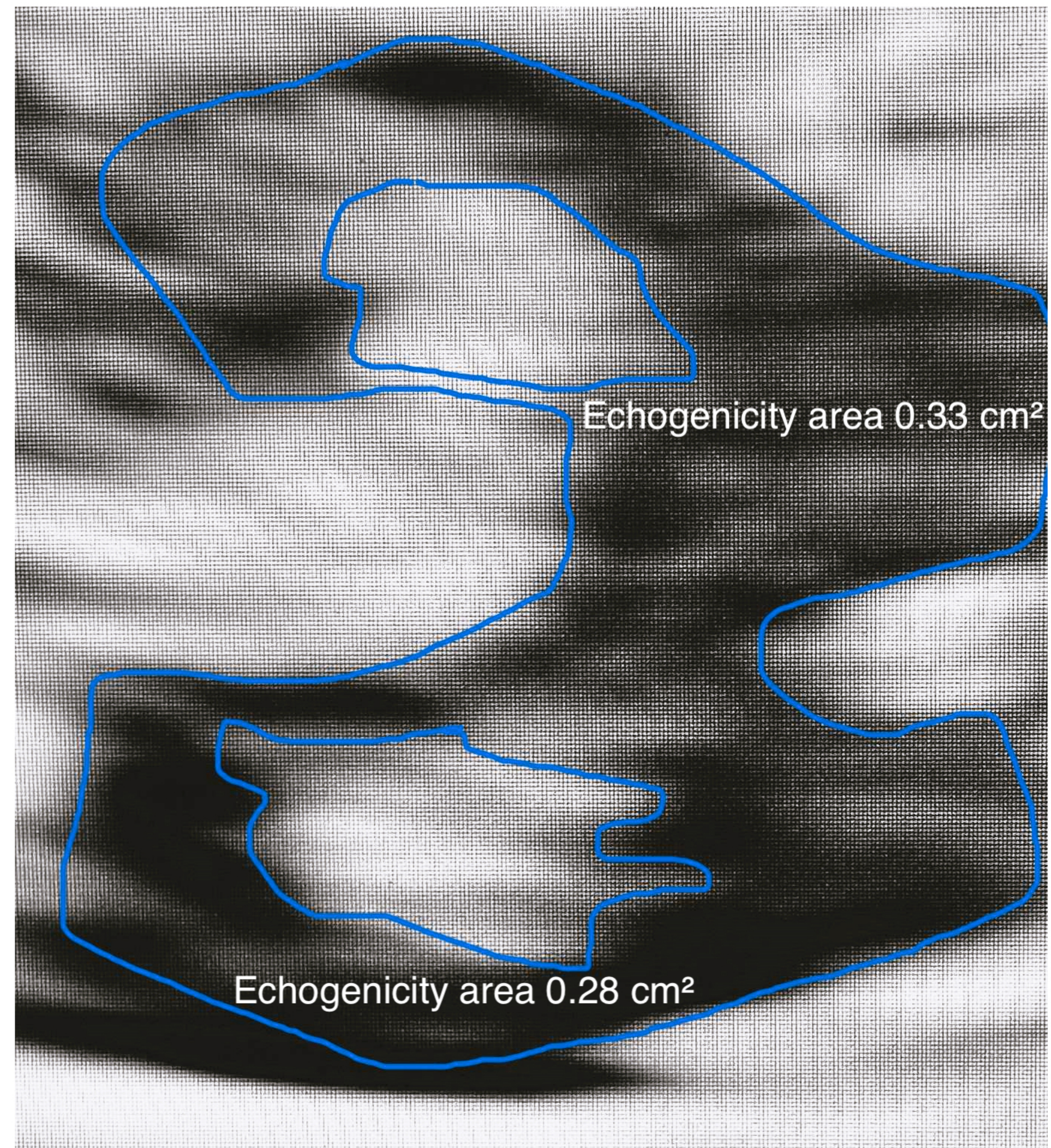**B**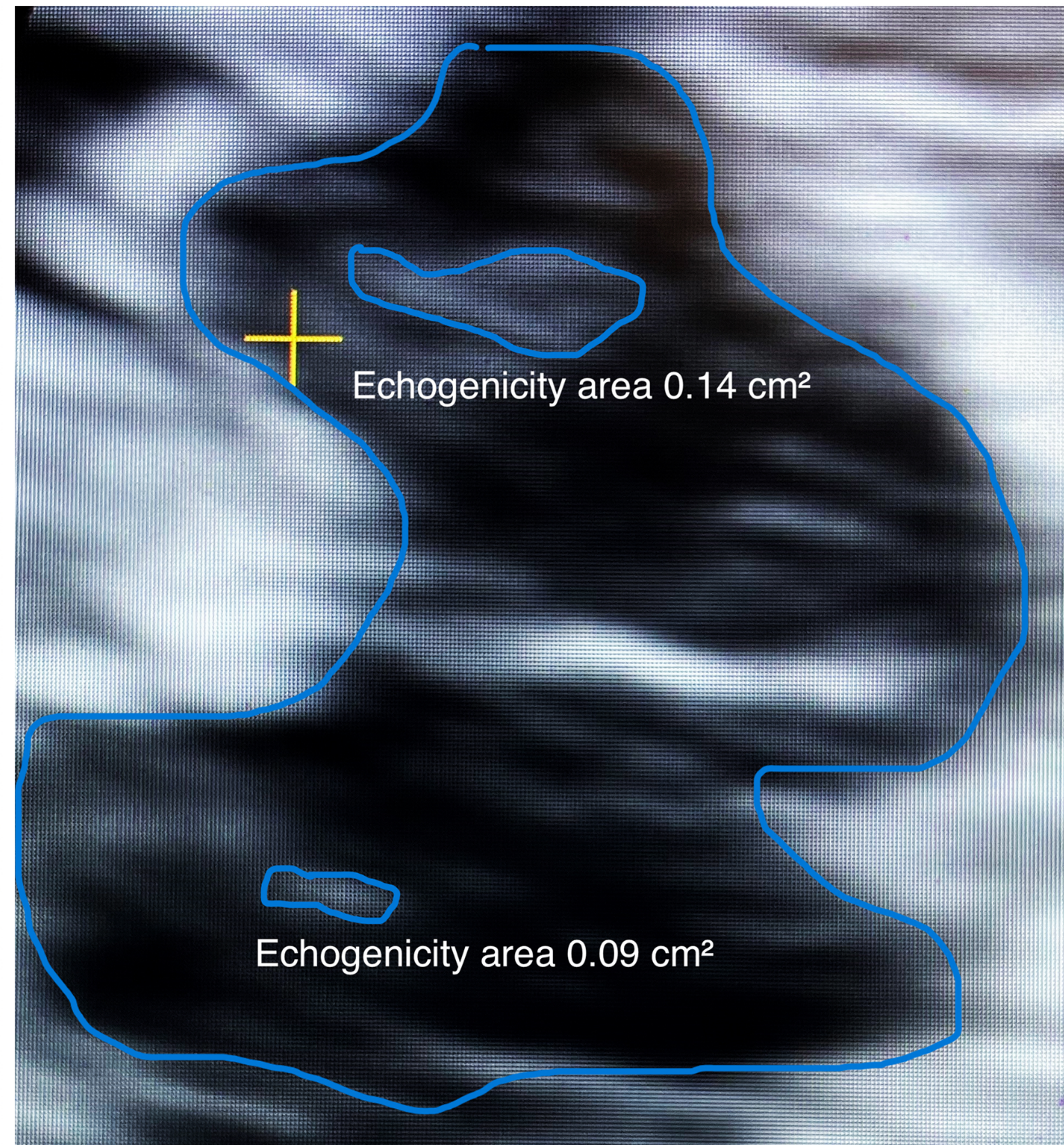

Supplement: Supplementary file 2 — Figure S2. Transcranial sonographic images of mesencephalic brain stems. (A) Image showing an individual with substantia nigra (SN) hyperechogenicity. (B) Image showing an individual without SN hyperechogenicity. The boundaries of the SN echogenicity areas were manually outlined, and numerical measurements were obtained using the TCS machine (ZST+ platform). [file MDC3-12-928-s006.pdf]

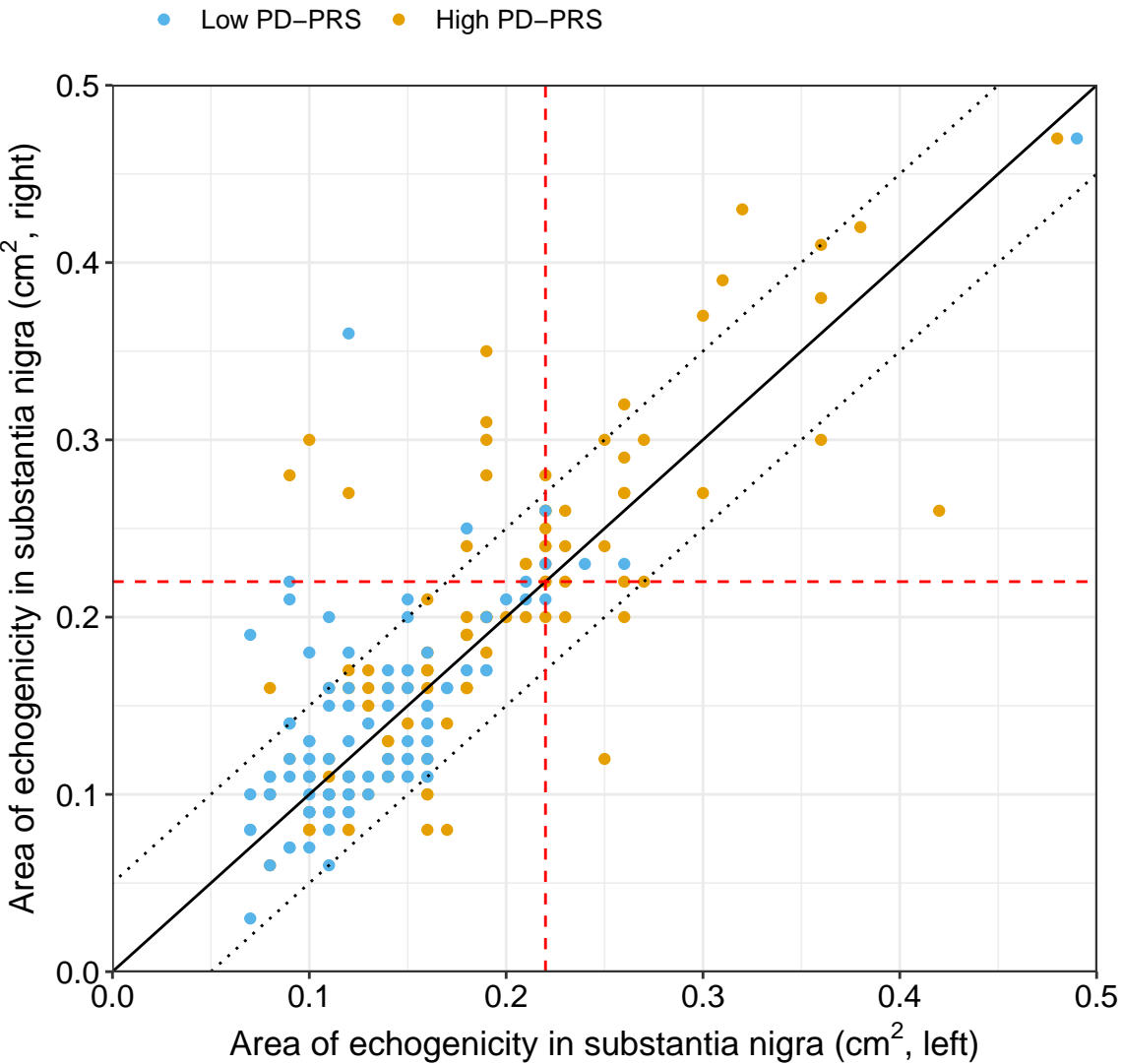

Supplement: Supplementary file 3 — Figure S3. Echogenicity areas of the substantia nigra (SN) measured from left and right temporal windows, stratified by polygenic risk score for Parkinson's disease (PD‐PRS) groups. The mean echogenicity values are 0.16 cm2 (standard deviation [SD]: 0.07) for the left side, 0.17 cm2 (SD: 0.08) for the right side, and 0.18 cm2 (SD: 0.08) for the maximum of both sides. A correlation analysis shows a strong association between left and right side measurements (Pearson's r = 0.80, P < 0.001). The solid line represents a slope of 1, with dotted lines marking ±0.05 cm2 intervals. The red dashed lines indicate the SN hyperechogenicity threshold value of 0.22 cm2. [file MDC3-12-928-s004.pdf]

Area of echogenicity in substantia nigra (cm<sup>2</sup>): ■ <0.22 ■ ≥ 0.22

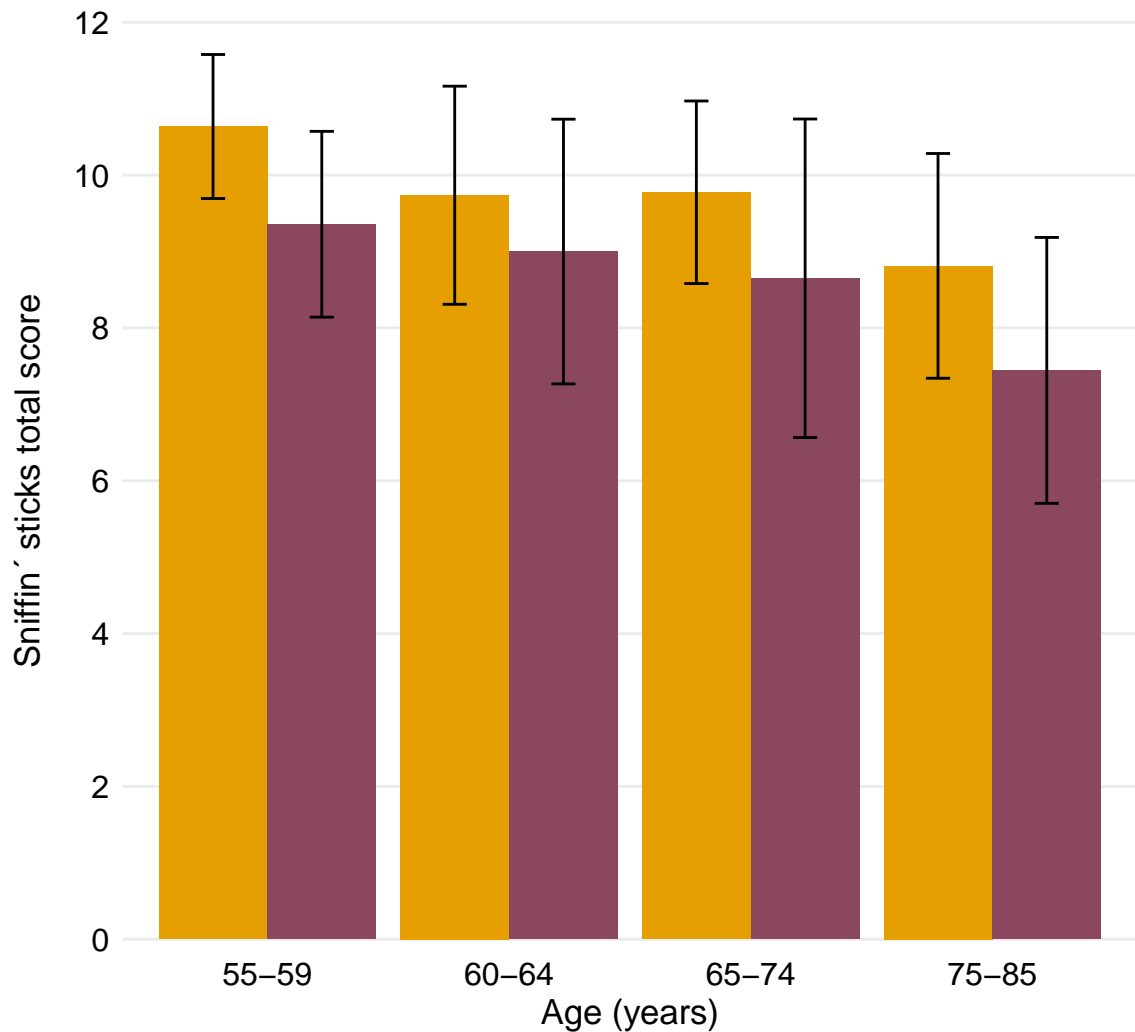

Supplement: Supplementary file 4 — Figure S4. Distribution of Sniffin’ sticks total score by age and substantia nigra (SN) echogenicity. Sniffin’ sticks scores decrease with advancing age and are significantly lower in individuals with SN hyperechogenicity (Pearson's χ2 test, both P < 0.001). Error bars represent ± standard deviations, illustrating the uncertainty in measurements. [file MDC3-12-928-s005.pdf]
