# Supplementary material for: Polygenic Risk Score Combined with Transcranial Sonography Refines Parkinson's Disease Risk Prediction
Source: Mov Disord Clin Pract. 2025 Feb 28;12(7):928–37. doi: 10.1002/mdc3.70011 (PMC12274997; doi:10.1002/mdc3.70011)
Supplement: Supplementary file 5 — Table S1. Sensitivity analysis excluding individuals with newly diagnosed Parkinson's disease (PD). Twelve individuals newly diagnosed with PD were excluded from the analysis. Participants were compared based on the presence (substantia nigra (SN) echogenicity area ≥0.22 cm2) or absence (SN echogenicity area <0.22 cm2) of SN hyperechogenicity. [file MDC3-12-928-s003.docx]

**Supplementary Table 1. Sensitivity analysis excluding individuals with newly diagnosed Parkinson’s disease (PD).** Twelve individuals newly diagnosed with PD were excluded from the analysis. Participants were compared based on the presence (substantia nigra (SN) echogenicity area ≥ 0.22 cm²) or absence (SN echogenicity area < 0.22 cm²) of SN hyperechogenicity.

|  | **SN**^*^ **≥** **0.22 cm^2^**  **(*n* = 44)** | **SN**^*^ **< 0.22 cm^2^**  ***(n* = 148)** | ***p*-value** |
| --- | --- | --- | --- |
| Age (SD, years) | 65.1 (7.0) | 64.6 (7.3) | 0.667 |
| Sex (%) |  |  | 0.334 |
| - female | 18 (40.9) | 75 (50.7) |  |
| - male | 26 (59.1) | 73 (49.3) |  |
| Family history of PD (%) | 5 (11.4) | 13 (8.8) | 0.825 |
| PD-PRS risk (%) |  |  | <0.001 |
| - high | 37 (84.1) | 61 (41.2) |  |
| - low | 7 (15.9) | 87 (58.8) |  |
| Sniffin’ sticks total score (SD) | 8.88 (1.92) | 9.92 (1.33) | <0.001 |
| Hyposmia (%) | 23 (54.8) | 47 (32.0) | 0.012 |
| Anosmia (%) | 4 (9.5) | 1 (0.7) | 0.009 |
| Dream enactment behavior (%) | 3 (6.8) | 14 (9.5) | 0.811 |
| Constipation (%) | 4 (9.1) | 20 (13.5) | 0.604 |
| Depression (%) | 7 (16.3) | 38 (25.9) | 0.274 |
| Diabetes (%) | 2 (4.5) | 15 (10.1) | 0.399 |
| Migraine with aura (%) | 5 (11.4) | 3 (2.0) | 0.022 |

^*^SN echogenicity area

SN, substantia nigra; SD, standard deviation; PD, Parkinson’s disease; PRS, polygenic risk score.
