# Supplementary material for: Polygenic Risk Score Combined with Transcranial Sonography Refines Parkinson's Disease Risk Prediction
Source: Mov Disord Clin Pract. 2025 Feb 28;12(7):928–37. doi: 10.1002/mdc3.70011 (PMC12274997; doi:10.1002/mdc3.70011)
Supplement: Supplementary file 6 — Table S2. Multivariable logistic regression analysis for substantia nigra (SN) hyperechogenicity. The analysis excludes 12 individuals with newly diagnosed Parkinson's disease, comparing participants with SN hyperechogenicity (SN echogenicity area ≥0.22 cm2, n = 44) and those without (SN echogenicity area <0.22 cm2, n = 148). [file MDC3-12-928-s001.docx]

**Supplementary Table 2. Multivariable logistic regression analysis for substantia nigra (SN) hyperechogenicity.** The analysis excludes 12 individuals with newly diagnosed Parkinson’s disease, comparing participants with SN hyperechogenicity (SN echogenicity area ≥ 0.22 cm^2^, *n* = 44) and those without (SN echogenicity area < 0.22 cm^2^, *n* = 148).

| **Model 1** | **OR (95% CI)** | ***p*-value** |  |
| --- | --- | --- | --- |
| Age (5-year increase) | 0.86 (0.66–1.12) | 0.265 |  |
| Sex: female | 0.85 (0.40–1.77) | 0.659 |  |
| Sniffin’ sticks total score | 0.63 (0.49–0.81) | <0.001 |  |
| *R^2^* = 12.1%, AUC = 0.660 (95% CI 0.558–0.762). | |  |  |
|  |  |  |  |
| **Model 2** | **OR (95% CI)** | ***p*-value** |  |
| Age (5-year increase) | 0.90 (0.67–1.20) | 0.483 |  |
| Sex: female | 0.77 (0.35–1.71) | 0.528 |  |
| Sniffin’ sticks total score | 0.65 (0.49–0.85) | 0.002 |  |
| PD-PRS risk: low | 0.14 (0.06–0.34) | <0.001 |  |
| *R^2^* = 28.4%, AUC = 0.796 (95% CI 0.720–0.871).  Likelihood-ratio test of model 2 vs. model 1, *p* < 0.001. | | | |

OR, odds ratio; CI, confidence interval; AUC, area under the receiver operating characteristic curve; PD, Parkinson’s disease; PRS, polygenic risk score.
